# *Primum non nocere* – Are chloroquine and hydroxychloroquine safe prophylactic/treatment options for SARS-CoV-2 (covid-19)?

**DOI:** 10.11606/s1518-8787.2020054002631

**Published:** 2020-06-26

**Authors:** Claudia Biguetti, Mauro Toledo Marrelli, Marco Brotto

**Affiliations:** I University of Texas-Arlington College of Nursing & Health Innovation Bone-Muscle Research Center ArlingtonTX USA University of Texas-Arlington. College of Nursing & Health Innovation. Bone-Muscle Research Center. Arlington, TX 76010, USA; II Universidade de São Paulo Faculdade de Saúde Pública Departamento de Epidemiologia São PauloSP Brasil Universidade de São Paulo. Faculdade de Saúde Pública. Departamento de Epidemiologia. São Paulo, SP, Brasil

**Keywords:** Coronavirus Infections, Chloroquine, toxicity, Hydroxychloroquine, toxicity, Contraindications, Drug

## Abstract

Chloroquine (CQ) and its analog hydroxychloroquine (HCQ) were recently included in several clinical trials as potential prophylactic and therapeutic options for SARS-COV-2 infection/covid-19. However, drug effectiveness in preventing, treating, or slowing the progression of the disease is still unknown. Despite some initial promising *in vitro* results, rigorous pre-clinical animal studies and randomized clinical trials have not been performed yet. On the other hand, while the potential effectiveness of CQ/HCQ is, at best, hypothetical, their side effects are factual and most worrisome, particularly when considering vulnerable groups of patients being treated with these drugs. in this comment, we briefly explain the possible mechanisms of action of CQ/HCQ for treating other diseases, possible actions against covid-19, and their potent side effects, in order to reinforce the necessity of evaluating the benefit-risk balance when widely prescribing these drugs for SARS-COV-2 infection/covid-19. We conclude by strongly recommending against their indiscriminate use.

## INTRODUCTION

SARS Cov-2 infection and Coronavirus disease (covid-19) brought the world to an unimaginable halt. Nevertheless, there is a strong need for up-to-date information about the evolution of the pandemic and the disease, the viability and testing of new antiviral therapies, and any drugs in consideration for prophylaxis and treatment of SARS-CoV-2/covid-19. Among several alternatives, Chloroquine (CQ) and its analog Hydroxychloroquine (HCQ) gained special attention from governments after the publication of *in vitro* studies^1^ followed by limited clinical results^2^, with vast news dissemination, particularly in the US and Brazil.

CQ and HCQ are antimalarial agents and have been prescribed for treatment of autoimmune diseases (e.g. rheumatoid arthritis and lupus) for almost 70 years^3^_._ The prescription of CQ/HCQ in malaria is for prophylaxis and/or for when drugs accumulate in malaria-infected erythrocytes and interfere with the toxic heme formation during the parasite growth. In rheumatic diseases, CQ/HCQ might exert multiple anti-inflammatory effects, which are associated with the drug affinity to the autophagosomes and lysosomes of leukocytes^3^. CQ/HCQ could affect the autoantigen presentation of leukocytes along the lysosomal pathway in autoimmune diseases, by disrupting the MHCII pathway^4^. In addition, CQ and HCQ alter the production of potent pro-inflammatory cytokines, such as TNFα and IL6, by interfering with the endosomal pH during the activation of Toll-like receptors^3,4^.

### Mechanisms of Action of CQ/HCQ on SARS-CoV-2/covid-19 Infection

In the context of the new SARS-CoV-2 infection and covid-19 pathogenesis, Figure shows possible mechanisms of action based on SARS (Severe Acute Respiratory Syndrome)^5^. For SARS-CoV-2, both CQ and HCQ perform antiviral activity in entry and post-entry stages of the 2019-nCoV in kidney epithelial cells (Vero E6 cell, ATCC-1586)^1,6^. SARS-CoV-2 uses the ACE2 (Angiotensin-converting enzyme-2) receptor for cell entry by receptor-mediated endocytosis, similarly to SARS-CoV. The entry of coronavirus into cell cytoplasm and the release of virus content depend on the endocytic machinery, which CQ/HCQ could disrupt due to its ability to neutralize the pH in these compartments^6^(Figure A). The immunomodulatory activity of CQ/HCQ might interfere with the strong inflammatory response triggered by SARS-Cov-2 during development of the infection, but these ideas are hypothetical and not confirmed in pre-clinical or clinical studies. In fact, a recent gold standard, randomized, double-blind, placebo-controlled trial across the United States and parts of Canada tested hydroxychloroquine as post exposure prophylaxis in 821 subjects. This trial reported that hydroxychloroquine did not prevent illness compatible with covid-19 or confirmed infection when used as post exposure prophylaxis within 4 days after exposure^7^. It has been demonstrated that the rapid virus infection and replication leads to a dysregulated immune response that progress to a cytokine storm response in severe covid-19, followed by the development of ARDS, septic shock, and eventual multiple organ failure^8^. Since CQ/HCQ might act through the inhibition of cell signaling mechanisms resulting in blunting of pro-inflammatory cytokines^9^(Figure B), it might be tempting to propose that both drugs would reduce the immunopathological tissue damage caused by viral infection^10^.

**Figure:**
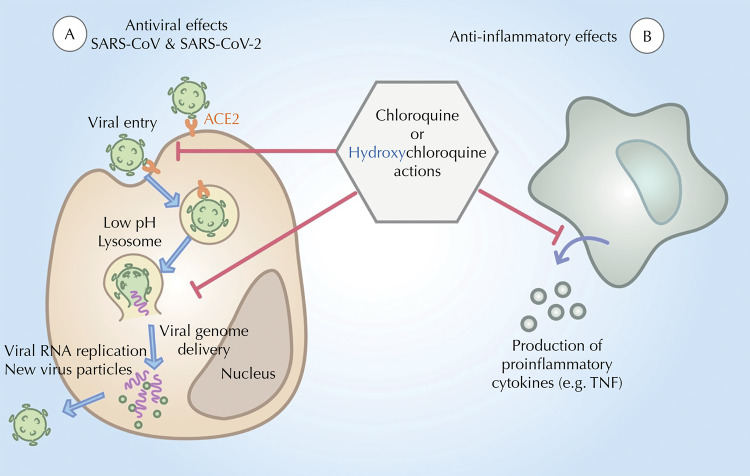
Possible mechanisms of action for CQ/HCQ on SARS-CoV-2/covid-19 Infection. A) *In vitro* antiviral activity of CQ and HCQ have been shown for SARS-CoV5 and SARS-CoV-21,6 viruses. Both drugs can inhibit viral entry and viral genome delivery to the cell cytoplasm, by interfering with the pH of endocytic pathway along with the formation of early endosomes and its maturation into a lysosome, respectively. B) Proposed anti-inflammatory role of CQ and HCQ against covid-19 inflammatory pathogenesis relies on the ability of the drug to inhibit the production of potent pro-inflammatory cytokines, such as TNFα and IL6, by interfering with the endosomal pH in leukocytes3,4.

### Toxic Effects of CQ/HQC

The major problem is that no rigorous pre-clinical cell-based, animal, nor randomized clinical studies were conducted to investigate these potential new mechanisms for antimalarial drugs or their clinical effectiveness to treat covid-19. However, there is bounteous evidence of their very detrimental side effects, and non-appropriated prescription can cause acute poisoning and even death. This is especially important when, in face of the recent spread of news for supposed benefits of CQ and HCQ against SARS-CoV-2/covid-19, individuals can self-medicate to prevent SARS-CoV-2 infection with off-label medicines, or obtain prescriptions from unethical practitioners. On March 31, 2020, The World Health Organization published specific guidance about the use of “off-label” drugs for covid-19 in clinical trials, especially concerning the use of CQ and HCQ without proper medical prescription^11^. On April 22, 2020, the National Institutes of Health published a strong rebuttal against their use for the treatment of covid-19^12^. On May 26, 2020, the World Health Organization temporarily suspended CQ and HCQ use in the Solidarity Trial^13^.

Due to their lysosomal affinity, CQ and HCQ accumulate in cells from several tissues with consequent tissue injury in the liver^14^, retina^15^, skeletal^16^, and cardiac muscle cells^17^. As announced by the FDA on April 7, 2020, side effects of HCQ include irreversible cardiac effects (including cardiomyopathy and QT prolongation), proximal myopathy and neuropathy^18^. Importantly, CQ and HCQ have long half-lives after oral administration (40-50 days)^3^. The resolution of cardiac and skeletal muscle symptoms is frequently slow after HCQ or CQ discontinuation^19^. Patients with CQ- or HCQ-induced myopathy frequently present progressive proximal muscle weakness, dyspnea, absent leg reflexes, and ventilator failure in severe cases^16,19,20^. Specifically for HCQ-induced cardiotoxicity, higher risk groups are older adults, cardiovascular patients, and patients with renal insufficiency^3^. Moreover, reported side effects of CQ/HCQ on skeletal and cardiac muscles are abundant for patients suffering from rheumatic diseases receiving these medications^3^. The US Center for Diseases Control and Prevention (CDC) recently updated the nine key symptoms for SARS-CoV-2/covid-19, and among these, three are related to the musculoskeletal system. Therefore, to prescribe prophylactic drugs that can trigger these problems in populations such older adults or obese/diabetic patients that have sub-optimal musculoskeletal health^21^ defies scientific reasoning.

### Final Considerations

The concept of using CQ/HCQ as prophylactic or therapeutic alternatives for SARS-Cov-2 infection is, at best, hypothetical, but their side effects are factual. In fact, CQ/HCQ could contribute to the exacerbation of musculoskeletal diseases in older adults at risk for developing severe covid-19^22^. Also, some of the characteristics of rheumatic patients at risk for developing CQ- and HCQ-induced myopathies are old age and other remarkable underlying medical conditions^3,23^, which are also found at the higher-risk patients for developing severe covid-19^2^. Thus, we end this comment by invoking the foundation of medical ethical treatment for over 2,500 years: *primum non nocere*– first do no harm!
